# circ2LO: Identification of CircRNA Based on the LucaOne Large Model

**DOI:** 10.3390/genes16040413

**Published:** 2025-03-31

**Authors:** Haihao Yu, Yue Yu, Yanling Xia

**Affiliations:** 1 Computer Science and Technology College, Heilongjiang Institute of Technology, No. 999 Hongqi Street, Harbin 150009, China; yuhaihao@hljit.edu.cn; 2 College of Animal Science, Jilin University, No. 1977 Xinzhu Road, Changchun 130012, China; yuyue9922@mails.jlu.edu.cn; 3 College of Wildlife and Protected Area, Northeast Forestry University, No. 26 Hexing Road, Harbin 150040, China

**Keywords:** circRNA, deep learning, large language model, transformer, self-attention mechanism

## Abstract

Circular RNA is a type of noncoding RNA with a special covalent bond structure. As an endogenous RNA in animals and plants, it is formed through RNA splicing. The 5′ and 3′ ends of the exons form circular RNA at the back-splicing sites. Circular RNA plays an important regulatory role in diseases by interacting with the associated miRNAs. Accurate identification of circular RNA can enrich the data on circular RNA and provide new ideas for drug development. At present, mainstream circular RNA recognition algorithms are divided into two categories: those based on RNA sequence position information and those based on RNA sequence biometric information. Herein, we propose a method for the recognition of circular RNA, called circ2LO, which utilizes the LucaOne large model for feature embedding of the splicing sites of RNA sequences as well as their upstream and downstream sequences to prevent semantic information loss caused by the traditional one-hot encoding method. Subsequently, it employs a convolutional layer to extract features and a self-attention mechanism to extract interactive features to accurately capture the core features of the circular RNA at the splicing sites. Finally, it uses a fully connected layer to identify circular RNA. The accuracy of circ2LO on the human dataset reached 95.47%, which is higher than the values shown by existing methods. It also achieved accuracies of 97.04% and 72.04% on the Arabidopsis and mouse datasets, respectively, demonstrating good robustness. Through rigorous validation, the circ2LO model has proven its high-precision identification capability for circular RNAs, marking it as a potentially transformative analytical platform in the circRNA research field.

## 1. Introduction

Due to the widespread application of high-throughput sequencing technology in recent years, tens of thousands of circular RNAs have been widely discovered in cells and tissues of various species. Research has found that circular RNA (circRNA) [1,2] plays important biological functions in cells through multiple pathways. Some circular RNAs act as competitive endogenous RNAs to regulate gene expression [3]. Circular RNA can also function as a molecular sponge to bind miRNA, thereby preventing the miRNA from inhibiting its target genes [4]. Some circular RNAs are even capable of being translated into proteins [5]. The study of circular RNA provides scientists with a novel perspective to comprehend the gene regulatory networks and cellular biological processes.

Circular RNA is closely related to the occurrence and development of various complex diseases, including Alzheimer’s disease, atherosclerosis, diabetes, and cancer. Their stability and tissue-specific expression make them potential disease biomarkers and therapeutic targets. For example, circular RNA can serve as a vaccine antigen carrier, providing a more durable immune response. Accurate identification of circular RNA is crucial for exploring the pathogenesis of human diseases and designing effective diagnostic and therapeutic strategies.

With the continuous development of high-throughput sequencing technology, a large number of short RNA read sequences have been measured and accumulated. Researchers have begun to use short RNA read sequences to identify circRNA [6,7] and have proposed several computational methods [8,9] for this task. These computational methods can be roughly divided into three categories: methods based on sequence fragment alignment, methods based on the reference genome, and methods based on machine learning. Methods based on sequence fragment alignment identify circRNA based on whether the short RNA read sequences contain back-splicing sites. For example, MapSplice [10] uses an accurate fragment alignment algorithm to detect multiple RNA splicing events, including back-splicing sites, and performs excellently in a complex transcriptome background. CIRCexplorer [11] focuses on the annotation of circRNA, combines back-splicing signals and multiple alignment methods to output data, and enables an in-depth analysis of the functions of circRNA. CIRI [12] identifies back-splicing signals through the maximum likelihood estimation method and combines multiple anchoring matching strategies to reduce false positives. Its improved version, CIRI2 [13], exhibits higher detection accuracy and running efficiency and supports the analysis of complex circRNA variants.

Methods based on the reference genome also use back-splicing sites as key clues in the identification of circRNA. For example, methods such as NCLscan [14], PTESFinder [15], and circall [16] are all based on the reference genome. These methods first construct candidate back-splicing sites through the reverse assembling of exons and then compare the assembled back-splicing sites with the reference genome sequence. If the constructed candidate back-splicing sites show a high matching degree with the reference genome sequence, these back-splicing sites are considered to exist, enabling the identification of the corresponding circRNA. However, the reference genomes of many species have not yet been systematically annotated. Furthermore, even the reference genomes of species with high research attention, such as humans and mice, are annotated from the perspective of traditional linear splicing rather than back-splicing. This severely limits the methods based on the reference genome to identify circRNA.

In recent years, machine learning has been applied to the identification of circRNA. Machine learning methods model the problem of circRNA identification as a binary classification problem. In this case, back-splicing sites are no longer the only clues for identifying circRNA. Instead, circRNA is characterized from multiple perspectives, and complex feature transformations are used to obtain the distribution characteristics of circRNA, based on which the potential circRNA is identified. Typical machine learning methods used for the task include DeepCirCode [17], CirNAPL [18], PredCirRNA [19], and circDeep [20]. Generally, these methods first collect circRNA and long noncoding RNA as positive and negative samples, respectively, then extract sample features from the sequence, structure, or genomic perspective, and, finally, use the sample features to train a classifier to identify circRNA. For example, DeepCirCode [17] uses a convolutional neural network to extract features from the back-splicing sites of circRNA and the upstream and downstream Alu repeat sequences, resulting in notably improved identification accuracy of circRNA and good prediction results in mammals. CirNAPL [18] adopts an extreme learning machine algorithm based on particle swarm optimization: it extracts 15 biological features from the composition and structure of RNA sequences and constructs an efficient classifier to effectively distinguish circRNA from other noncoding RNAs. PredCirRNA [19] combines multiple biological features, including sequence, structure, and upstream and downstream functional information, to construct a hybrid feature model. Compared to the previous two types of methods, the machine learning methods exhibit higher efficiency and a lower cost. However, they pay less attention to the structural differences between circRNA and other RNAs [21] and often require the use of additional feature extraction tools when extracting sample features, which affects the generalization performance of the models. Moreover, the direction, mismatch, and repetitive sequences of short read sequences can cause misjudgment of the back-splicing sites, resulting in relatively high false positives in the prediction results. The fundamental issue underlying these challenges stems from the limited representational capacity and insufficient robustness of the extracted RNA features, which fail to effectively capture the intricate patterns and nuanced variations present in RNA sequences.

To effectively solve the above problems, the present study proposes a method for the identification of circRNA based on the LucaOne [22] large model, named circ2LO, aiming to utilize the powerful feature extraction and generalization abilities of the above model to accurately identify circRNA. Specifically, in the circ2LO method, first, LucaOne is used for feature embedding of the splicing sites of RNA sequences and their upstream and downstream sequences to prevent semantic information loss caused by the traditional one-hot encoding method. Then, a convolutional layer is employed to extract features, and the self-attention mechanism is utilized to extract interactive features to accurately capture the core features of circRNA at the splicing sites. Finally, a fully connected layer (FCL) is used for decision-making to identify circRNA.

## 2. Methods and Models

Herein, we propose a method for the identification of circRNA based on the LucaOne large model. To construct circ2LO, Python 3.9 and PyTorch 2.5.1 were used. The source code for this project has been released on GitHub and is accessible at: https://github.com/Hhao2025/circ2LO (accessed on 22 March 2025).

**Figure 1:**
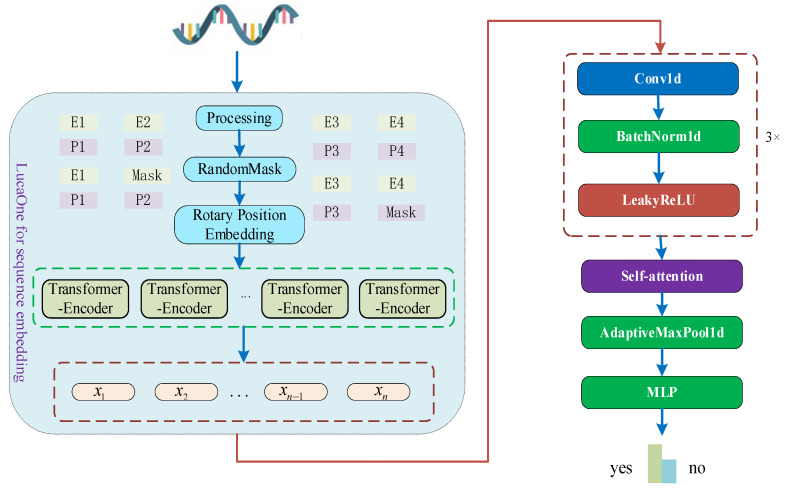
Overall model structure of the proposed method for circRNA identification based on the LucaOne large model. $x_{1},x_{2}\cdots x_{n-1},x_{n}$ denote an embedded representation of each nucleotide in an RNA sequence.

The structure of this method is shown in Figure 1. The proposed model consists of a sequence embedding module employing the LucaOne large model, a convolution module, a self-attention layer, an adaptive pooling layer, and a FCL.

### 2.1. RNA Sequence Embedding

**Figure 2:**
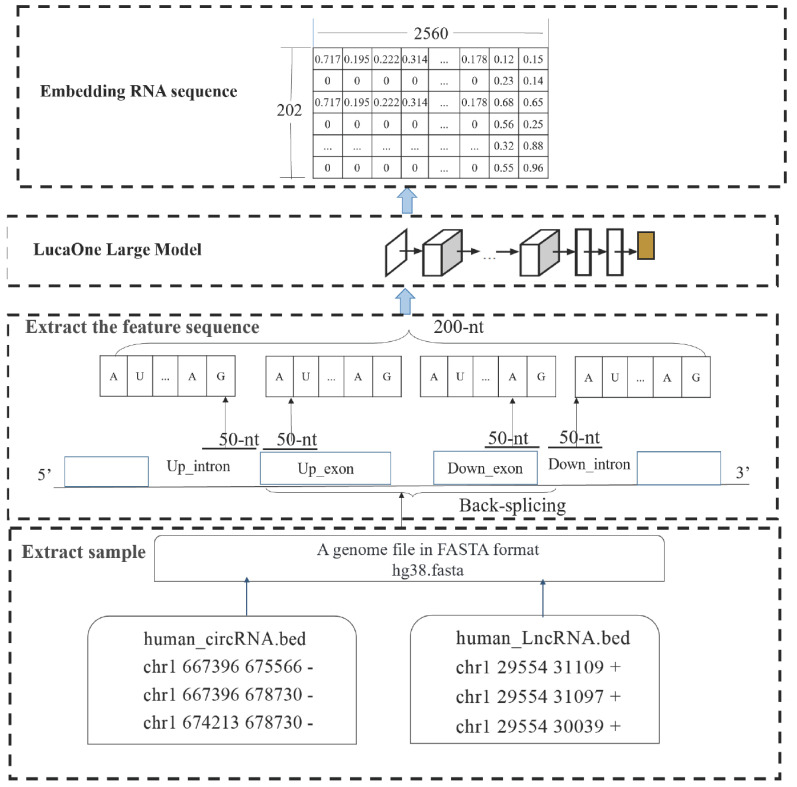
Implementation process, from metadata to obtaining the vector representation of RNA sequence embeddings.

RNA sequence embedding refers to the process of converting each nucleotide in an RNA sequence into a vector representation within a high-dimensional space. These vectors, or embeddings, are capable of capturing the contextual information of nucleotides within the sequence, as well as their roles in biological processes. The implementation process, from metadata to obtaining the vector representation of RNA sequence embeddings, is illustrated in Figure 2.

First, the bed file contains gene location information (for example, human circular RNA data are stored in the file human_circRNA.bed, while human long noncoding RNA data are stored in human_LncRNA.bed, as shown in Figure 2). Based on the start and end positions of the RNA recorded in these files, the nucleotide sequences of the corresponding RNA can be extracted.

Next, 50-nt sequences flanking each of the back-splicing sites are extracted, and the two 100-nt sequences are merged to form a full-length 200-nt feature sequence.

Finally, the extracted 200-nt feature sequence is used as input for LucaOne, where it is processed to generate a 202 × 2560 embedding RNA sequence.

The embedded RNA sequence, expressed as $x=\left[x_{1},x_{2},x_{3}\cdots ,x_{n}\right]$, is used as an input for the convolution layer, where feature extraction is performed. Subsequently, interaction features are extracted through the self-attention model and then fed to the pooling layer to form a vector that is used as an input for the FCL to classify whether the considered RNA sequence is circRNA.

### 2.2. LucaOne Large Model

In recent years, large language models have shown good potential for applications in bioinformatics because of their powerful feature extraction and generalization capabilities; they have been employed in various fields, including drug–target prediction [21,23,24,25], drug interaction prediction [26,27,28], and drug repositioning [29,30,31]. These models have driven innovation and progress in these fields by learning complex patterns of linguistic and biological sequences from massive data.

However, large language models have not yet been widely applied for the identification of circRNA. Herein, we aimed to leverage the powerful feature representation capabilities of large language models for the identification of circRNA. The splice sites of RNA sequences and their upstream and downstream regions contain complex and rich semantic information. However, traditional one-hot coding can only provide low-dimensional and sparse feature representations, complicating the capture of all the semantic information in the sequences and resulting in limited feature expression capability. In contrast, large language models are effective in processing complex sequence data. Through pretraining on large-scale biological sequence data, such models can learn rich contextual features, generate high-dimensional sequence embeddings containing semantic information, and, thereby, accurately capture the key features of splice sites, notably improving the recognition ability of circRNA.

To generate semantically rich RNA sequence embeddings, we directly processed RNA sequences using the LucaOne large model, the structure of which is shown in Figure 1. The LucaOne large model is pre-trained on nucleic acid and protein data to provide more efficient embedding representations for the prediction of biological sequence classifications. The LucaOne model is based on the transformer–encoder architecture and has been improved and optimized in various ways. The vocabulary consists of 39 unique tags to represent nucleotides and amino acids. The model employs rotated positional embedding instead of the traditional absolute positional encoding for longer sequences and employs tag-type embedding for the mixed training on nucleic acid and protein sequences. The LucaOne model consists of 20 transformer–encoder blocks, each containing 40 attention heads, supporting a maximum sequence length of 1280 and an embedding dimension of 2560. During embedding, the RNA sequences are first fed into the large model to generate context-sensitive embeddings, and then, these embeddings are used as inputs for the convolutional module for further feature extraction. In this paper, we did not optimize LucaOne. We only used this model to extract embeddings of RNA sequences, which were then processed by subsequent modules.

### 2.3. Convolution Module

Splice sites and their upstream and downstream sequences contain important feature information on the formation of circRNA. Herein, we used a convolutional module based on the LucaOne embedding representation to capture the local feature patterns of splice sites and their surrounding sequences, which provides robust feature representations for the accurate identification of circRNA. The model contains a total of three convolutional layers, each followed by a normalization layer and LeakyReLU activation functions. A normalization layer was introduced into the convolutional module to smooth out the variations in the distribution of splice sites and upstream and downstream features in order to ensure that the convolutional module and the downstream self-attention module can efficiently capture the key patterns and perform more consistently on data with high input diversity. LeakyReLU, which is different from the traditional ReLU activation function, was used as the activation function in the convolution module. When the input is less than 0, LeakyReLU outputs not a value of 0 but a small negative slope multiplied by the input value. This design enables the gradient transfer of the negative value to a certain extent, preventing the problem of neuron death encountered when using ReLU, and helps mitigate the problem of a vanishing gradient.

### 2.4. Self-Attention Layer

The attention mechanism [32] effectively improves the predictive performance of the model in various tasks by focusing on the most relevant features or regions in the data; the attention mechanism is widely used in the fields of drug–target interactions [33,34], drug–drug interactions [35,36], and drug side effect prediction [37,38]. Herein, we aimed to improve the recognition accuracy by characterizing the complex interactions between the RNA sequence information and splice sites through the attention mechanism. The attention mechanism can explicitly model the internal interactions of a sequence by calculating the correlation between the positions in the sequence, thus making it an ideal method for capturing these critical features. The formation of circRNA involves a specific back-splicing mechanism, with its core features distributed at the splice site and its upstream and downstream regions. While traditional fixed-window feature extraction methods may ignore remote dependencies, the attention mechanism can flexibly capture the interrelationship between any two points in the sequence, preventing information loss. In addition, certain positions in the RNA sequence, such as specific nucleotide combinations or signaling regions, may be critical for the formation of circRNA. The attention mechanism can dynamically focus on these important locations through adaptive weight assignment, thus enhancing the accuracy of feature extraction.

**Figure 3:**
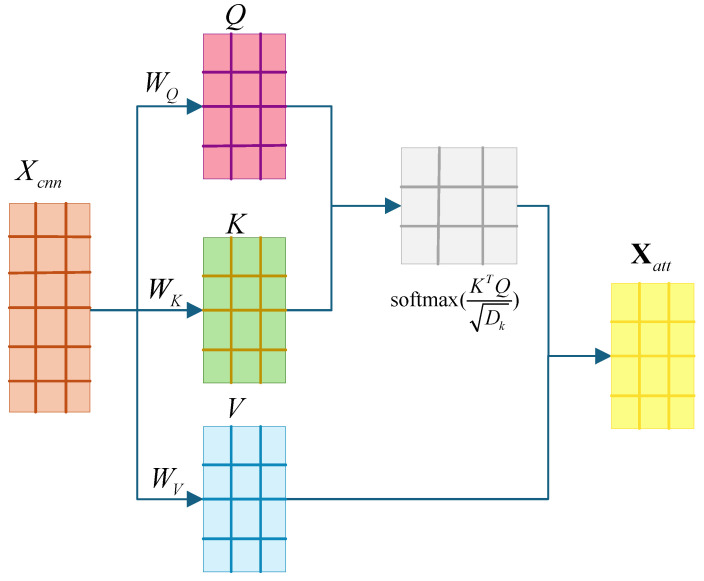
Schematic of the attention mechanism.

As shown in Figure 1, the model proposed herein applies the attention mechanism to the RNA features ($X_{cnn}$) after the convolutional layer to extract the contextual information from the sequence as well as the complex interaction features of the splice sites. The computation of attention is shown in Figure 3. First, the attention mechanism maps the input features ($X_{cnn}$) to Query, Key, and Value through independent linear transformations, which are calculated as follows:

(1) $$\begin{array}{l} Q=X_{cnn}W_{Q}\\ K=X_{cnn}W_{K}\\ V=X_{cnn}W_{V} \end{array}$$

where Q is the query vector, K is the key vector, V is the value vector, and W is the learnable weight matrix. Then, the similarity between each query vector and all the key vectors is calculated, normalized using the softmax function, and multiplied by the value vector. The corresponding formula is shown below:

(2) $$X_{att}=\mathrm{softmax}(\frac{K^{T}Q}{\sqrt{D_{k}}})V$$

where $X_{att}$ represents the interaction feature and $D_{k}$ represents the feature dimension. Attention enables the model to understand RNA sequence features more comprehensively by capturing the interaction patterns between features in the feature space, providing richer information for the recognition of circRNA. In this paper, we adjusted the number of heads in the self-attention layer to achieve the best performance.

### 2.5. Pooling Layer

After the extraction of the interaction features using the attention mechanism, an adaptive maximum pooling layer was utilized to select the most salient of the input features to further extract critical information. Maximum pooling can efficiently extract the most salient parts of the sequence with the most prominent feature values, which is especially important in the identification of circRNA because the key features may be confined to the vicinity of splice sites or certain high-confidence regions, and maximum pooling can capture these strong signals while ignoring the background noise. Compared to average pooling, maximum pooling is more sensitive to salient information in the features and helps the model focus on the core features in loop RNA recognition. Therefore, the adaptive maximum pooling layer can help the model extract the most important information and improve the robustness of the features. The input feature of the adaptive pooling layer is $X_{att}$, which is calculated as follows:

(3) $$Y\left[j\right]=\underset{i\in \left\{0,1,\ldots ,L-1\right\}}{\max}X_{att}\left[i,j\right]$$

where Y is the output feature after pooling.

### 2.6. Fully Connected Layer (FCL)

The pooling layer is followed by the FCL, where the pooled features are fused and classified using linear transformations and nonlinear activation functions. The FCL is a basic layer type in artificial neural networks, usually placed at the end of architectures such as convolutional neural networks. Neurons in a FCL are connected to all neurons in the previous layer, hence the name. It maps the distributed feature representations extracted in the previous layer to the decision space to transform the high-dimensional feature representation (Y) into a distribution associated with the target category to determine whether the input sequence is circRNA. Mapping is performed as follows:

(4) $$z=W\cdot Y^{T}+b$$

where W represents the weight matrix, b represents the bias matrix, and z represents the confidence that the input features belong to a certain category.

### 2.7. Loss Function

The loss function plays a crucial role in the training of the circRNA recognition model, where its main function is to measure the degree of similarity between the predicted values given by the neural network and the corresponding target values. By choosing an appropriate loss function, the training time and error can be considerably reduced, and the generalization ability of the model can be strongly improved. At present, cross-entropy loss is one of the most commonly used loss functions in classification problems; so, the cross-entropy loss function was adopted herein. The output of the model is $z\in R^{C}$, where C is the number of categories; in the case of circRNA recognition, this value is 2, indicating circRNA and non-circRNA. The logits are transformed into the category of probability distribution using the softmax function:

(5) $${\hat{y}}_{i}=\frac{e^{z_{i}}}{\sum_{j=1}^{2}e^{z_{j}}}$$

where ${\hat{y}}_{i}$ denotes the probability that the model’s prediction belongs to category i. The cross-entropy loss function is defined as follows:

(6) $$L=-\sum_{i=1}^{2}y_{i}\log\left({\hat{y}}_{i}\right)$$

During training, the model adjusts the model parameters by minimizing L so that the predicted probability ${\hat{y}}_{i}$ is as close as possible to the probability of the true category ($y_{i}$). This optimization of the loss function essentially maximizes the log-likelihood of the correct category predicted by the model, which in turn improves the classification performance.

## 3. Evaluation and Results

### 3.1. Experimental Datasets

To evaluate the performance of the circ2LO model, the circRNA and lncRNA sequences of humans, mice, and *Arabidopsis thaliana* were obtained from public databases such as circRNAdb, circRNAbase, and GENCODE and used as experimental datasets (Table 1). Among them, the human circular RNA data were obtained from two public databases: circRNADb [14] (version circRNAdb 1.0) and circBase [22] (version circBase 0.1). To improve the quality of the dataset, duplicate circRNAs from both databases were removed. A total of 92,369 entries were collected. Human lncRNA data were sourced from LNCipedia (version 5.1, available at https://lncipedia.org/download (accessed on 21 March 2025)), with a total of 111,557 entries collected. The human genome sequences in FASTA format were obtained from GENCODE (https://www.gencodegenes.org/human/release_21.html (accessed on 21 March 2025)). The mouse circular RNA data were obtained from a previous study by Werfel [22], with a total of 1903 entries collected. Mouse lncRNA data were sourced from the LncRBase database (http://bicresources.jcbose.ac.in/zhumur/lncrbase/index_download.html (accessed on 21 March 2025)), with a total of 1744 entries collected. The mouse genome sequences in FASTA format were obtained from GENCODE (https://www.gencodegenes.org/mouse/release_M19.html (accessed on 22 March 2025)). The Arabidopsis thaliana circular RNA data were obtained from PlantCircBase (http://ibi.zju.edu.cn/plantcircbase/view.php (accessed on 22 March 2025)), with a total of 52,393 entries collected. The *Arabidopsis thaliana* lncRNA data were sourced from PLncDB (http://plncdb.tobaccodb.org/SpeciesDetail?speciesName=Arabidopsis_thaliana&isSpecie=lncRNA (accessed on 23 March 2025)), with a total of 7887 entries collected. The *Arabidopsis thaliana* genome sequences in FASTA format were obtained from the TAIR database (https://www.arabidopsis.org/ (accessed on 23 March 2025)).

The human dataset includes 92,369 circRNA and 111,557 lncRNA data entries; the mouse dataset contains 1903 circRNA and 1744 lncRNA data entries; and the *Arabidopsis thaliana* dataset comprises 52,393 circRNA and 7887 lncRNA data entries. All subsequent performance evaluation experiments used these experimental datasets. In the experimental datasets, circular RNAs were used as positive samples, and long noncoding RNAs were used as negative samples. The experimental dataset is divided into a training set, a validation set, and a test set, with 80% used for training, 10% for validation, and 10% for testing.

### 3.2. Evaluation Method

Herein, accuracy, precision, recall, sensitivity, F1-score, and the Matthews Correlation Coefficient (MCC) were used to evaluate the performance of the model.

From a biological perspective, recall is crucial for minimizing false negatives, ensuring that genuine circRNAs are not overlooked, while precision is essential for reducing false positives, preventing the erroneous detection of circRNAs. The F1-score balances these two metrics, providing a single comprehensive measure of overall performance. A high MCC value indicates strong agreement between predicted and actual circRNAs, reflecting the model’s robustness and reliability in practical applications.

### 3.3. Comparative Experiment

Herein, we first used the human dataset to verify the comprehensive performance of the proposed method. As shown in Table 2, the performance of the proposed method on the human dataset was compared to those of existing advanced methods such as circDeep, CirRNAPL, DeepCirCode, PredcircRNA, and lncRNA-master. The results indicate that circ2LO exhibits notably higher values in all metrics. Compared to the best baseline model (PredcircRNA), it shows substantially higher accuracy, precision, recall, F1-score, and MCC, with increases of 5.32%, 5.31%, 5.27%, 5.31%, and 5.65%, respectively. This indicates that circ2LO is better at identifying circRNA and lncRNA samples, can classify samples more accurately, and gives fewer misjudgments. Notably, the MCC value (which is a comprehensive evaluation metric) of circ2LO reaches 90.95%, further confirming the excellent performance of the proposed method on the human dataset and further demonstrating the better overall performance of the model in distinguishing between circRNA and lncRNA. The proposed model not only performs well in a single metric but achieves a good balance and synergy among various metrics, enabling it to adapt to complex datasets and practical application scenarios. Such outstanding performance was attributed to the targeting of the splice sites and their upstream and downstream RNA sequences and the use of the LucaOne large model to deeply mine their potential semantic information and generate high-quality feature embedding vectors. This process effectively prevents semantic information loss caused by the traditional one-hot encoding method. Subsequently, the self-attention mechanism can dynamically focus on the correlation information between different positions in the sequence and accurately capture the core features at the circRNA splice sites. In human circRNA, the features of splice sites are often affected by their upstream and downstream sequences, and the self-attention mechanism can adaptively adjust the weights to highlight the contributions of the features at key positions, enabling the model to understand and identify the unique structural patterns of circRNA.

To comprehensively explore the generalization performance of the proposed method, the mouse dataset was selected for further verification. The mouse dataset is smaller than the human dataset. However, as a classic model, the data for mice contain the characteristic information on circRNA, which is scarce for mammals. Therefore, it is of crucial importance to verify whether the proposed method can efficiently identify circRNAs across species. Table 3 shows that the proposed method maintains high performance on the mouse dataset. Compared to the best baseline model (PredcircRNA), circ2LO exhibits improvements in all metrics. This indicates that after the feature embedding of the splicing sites and their upstream and downstream sequences of mouse RNA using the LucaOne large model, circ2LO can efficiently extract species-specific feature information based on the characteristics of the mouse gene sequences. After being processed by the convolutional layer and the self-attention mechanism, this feature information can accurately capture the key features at the splicing sites of mouse circRNA. Although there may be certain differences in the structure and function between mouse and human circRNA, the adaptive feature extraction ability of the proposed method enables the model to quickly adapt to the characteristics of the mouse dataset and accurately and efficiently identify circRNA.

The research on circRNA in plants is also of great significance. Thus, the data on *Arabidopsis thaliana* as a model plant were used for verifying the universality of the circ2LO method. As shown in Table 4, the advantages of the proposed method are also evident for the *Arabidopsis thaliana* dataset. The precision reaches 98.16%, which is 3.96% higher than that of the baseline model, PredcircRNA. This indicates that circ2LO can effectively detect circRNA, with a good balance among various performance indicators, and can adapt to the characteristics of the *Arabidopsis thaliana* dataset. These results indicate the strong generalization ability of the proposed method and highlight its potential for use in cross-species studies. The research on circRNA in plants is also of great significance. Thus, the data on *Arabidopsis thaliana* as a model plant were used for verifying the universality of the circ2LO method. As shown in Table 4, the advantages of the proposed method are also evident for the *Arabidopsis thaliana* dataset. The precision reaches 98.16%, which is 3.96% higher than that of the baseline model, PredcircRNA. This indicates that circ2LO can effectively detect circRNA, with a good balance among various performance indicators, and can adapt to the characteristics of the *Arabidopsis thaliana* dataset. These results indicate the strong generalization ability of the proposed method and highlight its potential for use in cross-species studies.

To validate the performance of our proposed method, we employed the model trained on human datasets to perform circRNA identification tests on *Drosophila* datasets. The *Drosophila* circular RNA and lncRNA data, as well as the genome sequences in FASTA format, were all sourced from the research conducted by Jun Wang [17]. The experimental results demonstrate that our model achieves an identification precision of 74.7%. This excellent recall performance confirms that our method can effectively identify circRNAs, demonstrating good cross-species applicability and strong competitiveness.

### 3.4. Ablation Experiment

Several model configurations, including circ2LO, circ2LO without the LucaOne macromodel, and circ2LO without the self-attention layer, were constructed to assess their performance metrics and examine the effects of important components, such as the self-attention mechanism and the LucaOne model, on the overall performance of circ2LO, thereby determining the viability and significance of each component. Furthermore, ablation experiments on the fundamental parameters of circ2LO were conducted.

**Figure 4:**
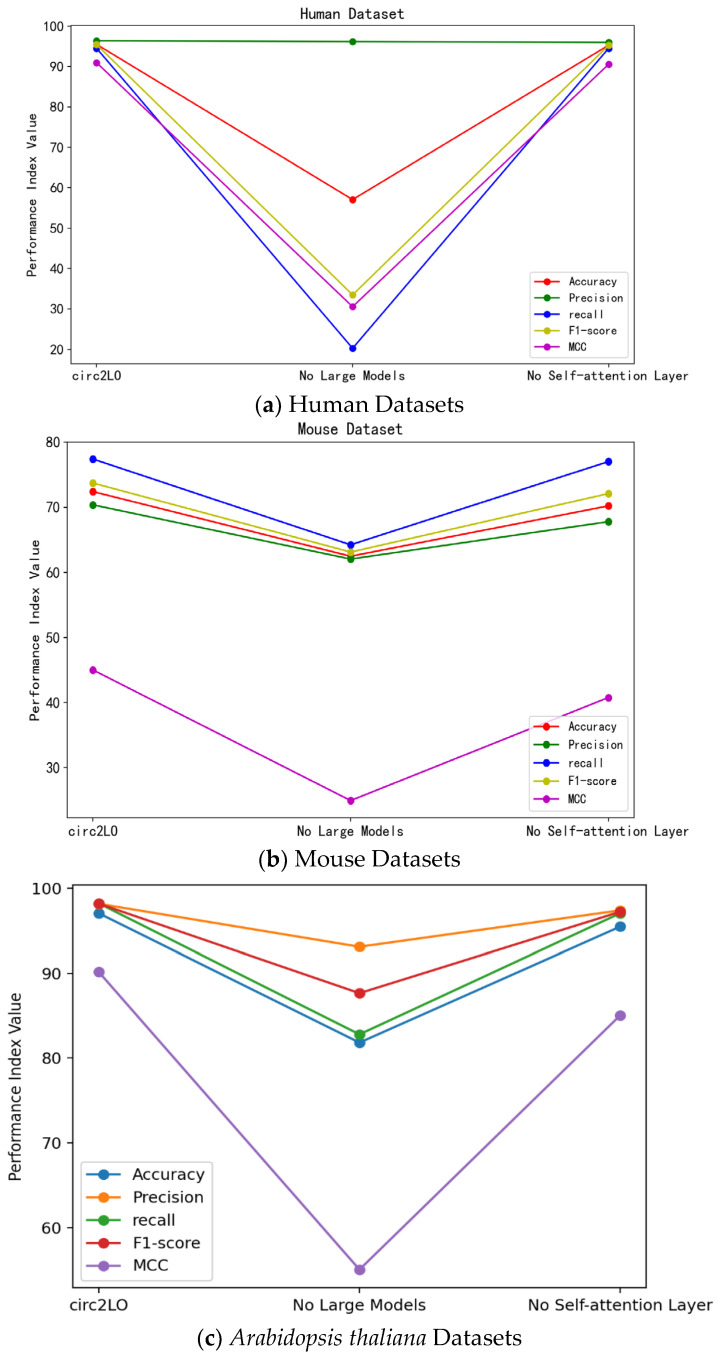
Results of the quantitative analysis of the key modules.

The results of quantitative analysis for each module are shown in Figure 4, indicating that the performance of circ2LO on the human dataset is determined by the use of LucaOne. In particular, circ2LO shows notably better performance than circ2LO without the LucaOne model. This implies that the good representational capacity of the LucaOne model improved the ability of circ2LO to match the data and, consequently, its accuracy.

Circ2LO exhibits a recall of 94.50%, whereas circ2LO without LucaOne shows a recall of only 20.28%. This further demonstrates that the use of the large language model makes the method more robust and helps identify positive cases.

On the *Arabidopsis thaliana* dataset, circ2LO shows an accuracy of 97.04%, whereas circ2LO without the self-attention layer shows an accuracy of 95.47%. This suggests that the self-attention mechanism has a fine-tuning effect on the final outcome. The MCC of circ2LO on the human dataset is 90.95%, which is marginally higher than the MCC of circ2LO without the self-attention layer. The MCC of circ2LO on the *Arabidopsis thaliana* dataset is 90.04%, whereas the MCC of circ2LO without the self-attention layer is 85.02%. These results suggest that the self-attention mechanism helps the model make more accurate judgments in the classification of positive and negative samples, leading to a higher correlation between the model’s predictions and the true labels.

### 3.5. Parametric Ablation Experiment

(1) Convolutional layer

**Figure 5:**
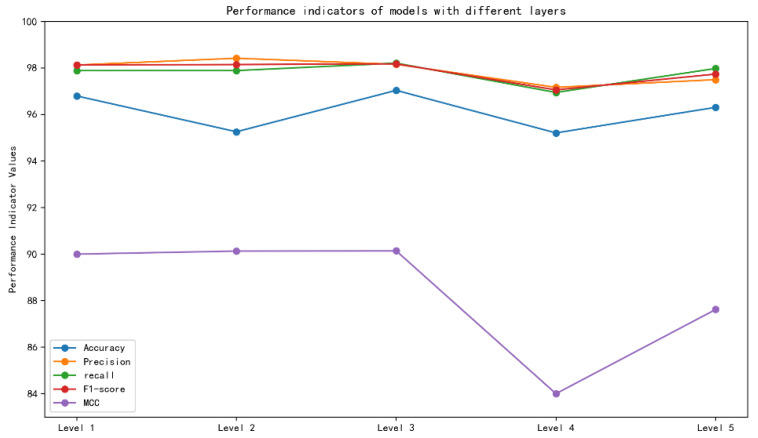
Effect of the number of convolutional layers on the model’s performance.

Figure 5 shows the effects of the number of convolutional layers on the model’s performance. The model with three convolutional layers ranks highly in terms of accuracy, precision, recall, and F1-score, and a high MCC demonstrates its strong performance in distinguishing between positive and negative samples. MCC comprehensively evaluates the classification performance of both positive and negative samples because it considers all four components of the confusion matrix. Consequently, the model with three convolutional layers was selected as the optimal solution.

(2) Effect of learning rate

**Figure 6:**
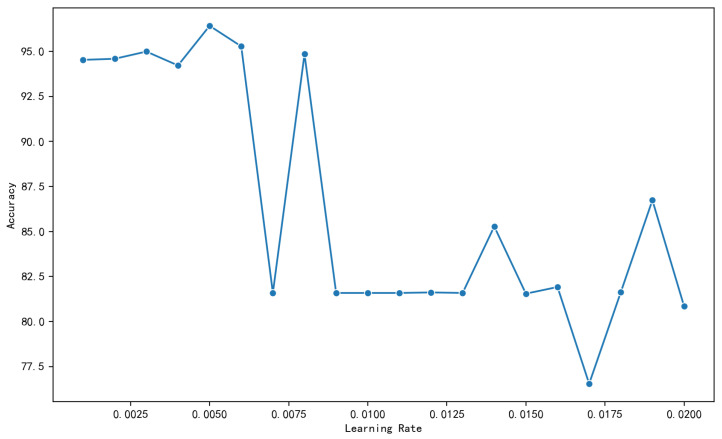
Effect of learning rate on model accuracy.

Figure 6 shows the effect of learning rate on accuracy. At low learning rates, accuracy fluctuates but remains high and relatively steady. Accuracy reaches 96.42% at a learning rate of 0.005, indicating a consistent upward trend. This is because the model can update the weights in the gradient direction of the loss function in smaller steps due to the low learning rate. The model may adjust the settings more precisely within this range. The accuracy falls to 81.59% at a learning rate of 0.007 and maintains this value at higher learning rates. This suggests that the learning rate range might be too wide, complicating the convergence to the optimal solution and causing the model to fluctuate as the weights are updated. Thus, 0.005 was considered the optimum learning rate.

(3) Linear layer

**Figure 7:**
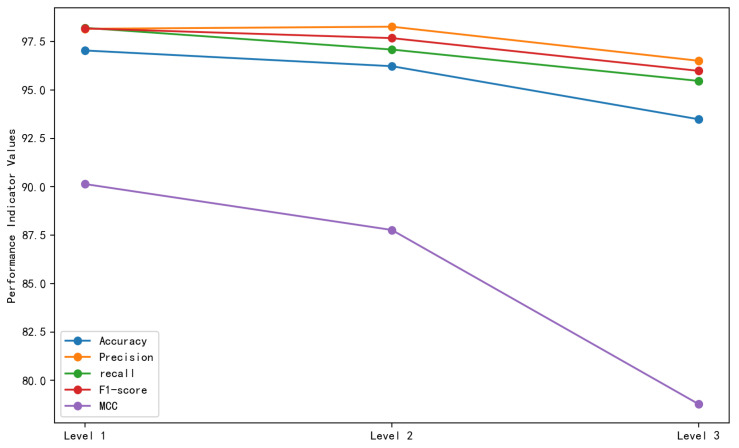
Effect of the number of linear layers on the model’s performance.

Figure 7 shows the effect of the number of linear layers on the model’s performance. The model with three linear layers exhibits the best accuracy (97.04%), indicating excellent performance of three linear layers in the classification of the samples. At the same time, the model with one linear layer shows the best performance when all performance parameters are taken into account. Thus, a single linear layer was considered optimal for the current experimental setup and task because it strikes a balance between feature mapping and decision boundary learning. It can also handle the input data effectively and performs well in classifying positive and negative samples.

## 4. Conclusions

Herein, we propose circ2LO, a method for the identification of circRNA based on the LucaOne large model. In circ2LO, the LucaOne large model is utilized to perform feature embedding of the splicing sites of RNA sequences as well as their upstream and downstream sequences to prevent semantic information loss caused by the traditional one-hot encoding method. Subsequently, features are extracted using a convolutional layer, and the interactive features are extracted through the self-attention mechanism to accurately capture the core features of circRNA at the splicing sites. Finally, a FCL is employed to identify circRNA. Herein, comparative experiments, ablation experiments, and multispecies experiments were conducted on multiple public datasets. The results indicate better comprehensive performance (accuracy, precision, recall, F1-score, and MCC) of the circ2LO model compared to previously reported circRNA identification models. In summary, the circ2LO model exhibits high accuracy and reliability in circRNA identification and is expected to become a powerful tool in the field of circRNA research.

Our proposed circ2LO can effectively capture complex sequence patterns and structural features, thereby improving the accuracy of circRNA prediction. However, real-world datasets often face challenges such as noise and incomplete data, which may affect the model’s performance. To address these future challenges, strategies such as transfer learning and robust model architectures can be employed to enhance generalization capabilities. Moreover, integrating biological knowledge can improve the model’s interpretability and reliability. Future improvements may also include developing ensemble models that combine multiple prediction strategies to enhance the model’s robustness and performance in practical applications.

## Figures and Tables

**Table 1:** Positive examples (circRNA) and negative examples (lncRNA) in experimental datasets.

Datasets	circRNA Count	lncRNA Count
Human	92,369	111,557
*Arabidopsis thaliana*	52,393	7887
Mouse	1903	1744

**Table 2:** Performance comparison on the human dataset.

Methods	Accuracy	Precision	Recall	F1-Score	MCC
circDeep	80.56	81.08	61.13	70.12	65.35
CirRNAPL	85.46	86.12	84.36	85.20	80.18
DeepCirCode	85.24	87.58	88.61	88.09	70.38
PredcircRNA	90.15	91.05	89.23	90.11	85.30
circ2LO	95.47	96.36	94.50	95.42	90.95

**Table 3:** Performance comparison on the mouse dataset.

Methods	Accuracy	Precision	Recall	F1-Score	MCC
circDeep	68.5	65.2	70.1	67.5	38.2
CirRNAPL	70.2	68.8	72.3	72.5	40.1
DeepCirCode	72.8	69.1	68.5	69.7	41.6
PredcircRNA	71.3	70.2	74.8	71.9	42.8
circ2LO	72.4	70.4	77.4	73.7	45.0

**Table 4:** Performance comparison on the *Arabidopsis thaliana* dataset.

Methods	Accuracy	Precision	Recall	F1-Score	MCC
circDeep	93.25	91.10	93.80	89.95	83.50
CirRNAPL	94.78	95.30	95.05	95.18	86.22
DeepCirCode	92.16	92.80	89.45	91.63	80.95
PredcircRNA	95.50	94.20	96.00	96.10	87.80
circ2LO	97.04	98.16	98.21	98.18	90.14

## Data Availability

The original contributions presented in this study are included in the article; further inquiries can be directed to the corresponding author.
